# Screening for aberrantly methylated and differentially expressed genes in nonalcoholic fatty liver disease of hepatocellular carcinoma patients with cirrhosis

**DOI:** 10.1186/s12957-022-02828-3

**Published:** 2022-11-18

**Authors:** Guizhi Zhang, Yinghui Hou

**Affiliations:** Department of Gastroenterology, The Second People’s Hospital of Liaocheng, No. 306, Health Street, Linqing, 252600 Shandong China

**Keywords:** Hepatocellular carcinoma, Liver cirrhosis, Nonalcoholic fatty liver disease, Methylation

## Abstract

**Background:**

Nonalcoholic fatty liver disease (NAFLD) as the leading chronic liver disease worldwide causes hepatic fibrosis, cirrhosis and hepatocellular carcinoma (HCC). The aim of this study was to find potential aberrantly methylated and differentially expressed genes in NAFLD of HCC patients with cirrhosis.

**Methods:**

DNA methylation data, mRNA expression data, and the corresponding clinical information of HCC were downloaded from the Cancer Genome Atlas (TCGA, tissue sample) database. HCC patients with cirrhosis were divided into two groups according to the presence of NAFLD. The differentially expressed genes (DEGs) and differentially methylated genes (DMGs) were obtained.

**Results:**

By overlapping 79 up-regulated genes and 1020 hypomethylated genes, we obtained 5 hypomethylated-highly expressed genes (Hypo­HGs). By overlapping 365 down-regulated genes and 481 hypermethylated genes, we identified 13 hypermethylated-lowly expressed genes (Hyper-LGs). Survival analysis of these 18 MDEGs indicated that the expression of DGKK and HOXD9 was significantly correlated with the overall survival time of NAFLD patients.

**Conclusions:**

We identified several candidate genes whose expressions were regulated by DNA methylation of NAFLD of HCC with cirrhosis, which may provide a new field in understanding the clinical pathological mechanism of NAFLD of HCC with cirrhosis.

## Background

Nonalcoholic fatty liver disease (NAFLD) is emerging as the leading chronic liver disease worldwide [18]. NAFLD, including nonalcoholic steatohepatitis (NASH), causes hepatic fibrosis, cirrhosis, and hepatocellular carcinoma (HCC) [9]. A population-based cohort (*n* = 8,892) study indicated that NAFLD patients had significantly increased overall cancer incidence, especially HCC, compared to the controls, adding evidence of cancer risk in patients with NAFLD [28]. The characteristics of patients with HCC, secondary to NAFLD, are older age, large tumors due to late diagnosis, often without cirrhosis, and high prevalence of the metabolic syndrome components, leading to an increased mortality rate [2].

HCC, the most prevalent form of liver cancer, is one of the most frequently occurring malignancy around the world [30]. HCC is an insidious tumor that is often diagnosed in the later stage of life [25]. Moreover, the HCC recurrence occurred in patients with hepatitis D virus when hepatitis B virus recurrence developed [3]. It is reported that Hepatitis B and C virus-related chronic liver disease have exceptionally high risk of HCC development [15, 29]. The epidemiologic data also support that HCC incidence is highest in regions with the higher incidence of HBV. In addition, cirrhosis and smoking are important risk factors for HCC. NAFLD has become one of the leading etiologies for HCC [24]. It is reported that NAFLD-related HCC tends to occur in older individuals and tends to be diagnosed at a later stage [38]. In addition, lifestyle, social conditions, and ethnicity may contribute to the incidence of NAFLD-related HCC [24]. Given the high incidence of NAFLD and its close correlation with the occurrence of HCC, it is of great significance to reveal the pathogenesis of NAFLD and the possible mechanism of its transformation to HCC.

DNA methylation has emerged as an important epigenetic modification and plays a key role in the regulation of gene expression and genome stability [5]. Alterations in DNA methylation in promoter regions has been reported to contribute to the occurrence, development, and prognosis of multiple cancers [7]. Accumulating evidence indicates that the dynamic patterns of DNA methylation are closely associated with the development, diagnosis, and prognosis of liver cancer [11, 22]. It has been reported that DNA methylation silenced BCLB gene expression participates in the progression of HCC, indicating its therapeutic implications for HCC [16]. In alcohol-related HCC, retinol metabolism genes and serine hydroxymethyltransferase 1 are epigenetically regulated via promoter DNA methylation [33]. Besides, Kuo et al. found that IRAK3 methylation was associated with the tumor stage and poor prognosis of HCC patients [10]. Therefore, DNA methylation alteration may play a coordinating role in promoting the carcinogenesis and progression of liver cancer.

In the present study, we aimed to evaluate methylation changes specific to NAFLD of HCC with cirrhosis that could be used as tools in the clinical setting for prognostic assessment of patients. To achieve this goal, we used publicly available microarray data to determine the differentially expressed genes (DEGs) and differentially methylated genes (DMGs) between non-NAFLD and NAFLD HCC patients with cirrhosis. Methylated differentially expressed genes (MDEGs) were obtained by overlapping DEGs and DMGs. Comprehensive and advanced bioinformatics analysis of existing microarray data can reveal more reliable and precise disease-related results.

## Materials and methods

### Data collection

The Cancer Genome Atlas (TCGA) database contains clinical information from tumor tissues, normal tissue, or blood samples from hundreds of patients with specific cancers on a large scale, which is used for comprehensive genomic data analysis and integration analysis. Currently, the TCGA database covers global information with more than 30 kinds of cancers, including genome variation, gene expression, copy number, genotypes, DNA methylation, and exon sorting. For HCC, a total of 377 patients with HCC were included in the TCGA database, including clinical data of 377 patients, RNA sequencing data of 371 patients, and methylation array data of 377 patients. The DNA methylation data, mRNA expression data, and the corresponding clinical information of HCC were downloaded from the TCGA database (involving tissue sample). According to the fibrosis score, 142 HCC patients with cirrhosis were selected. Then, 142 HCC patients with cirrhosis were divided into two groups according to the presence of NAFLD samples with both mRNA expression data and DNA methylation data included. Consequently, 126 non-NAFLD and 12 NAFLD HCC patients with cirrhosis were included in this study. Clinical information of patients included in this study is indicated in Table 1. Student’s *t*-test was performed for continuous variable, and chi-square test was performed for categorical variable.

**Table 1:** Summary of clinical information

	NAFLD (*n*=12)	Non-NAFLD (*n*=126)	*p*-value
Age (years, mean (SD))	61.00 (9.61)	59.10 (12.00)	0.595
Sex = male (%)	6 (50.0)	100 (79.4)	0.052
BMI (kg/m^2^, mean (SD))	32.18 (6.56)	26.36 (10.75)	0.069
T (%)			0.513
T1	8 (66.7)	73 (58.4)	
T2	4 (33.3)	32 (25.6)	
T3	0 (0.0)	17 (13.6)	
T4	0 (0.0)	3 (2.4)	
N (%)			0.434
N0	7 (58.3)	93 (74.4)	
N1	0 (0.0)	1 (0.8)	
NX	5 (41.7)	31 (24.8)	
M (%)			0.386
M0	7 (58.3)	95 (75.4)	
M1	0 (0.0)	1 (0.8)	
MX	5 (41.7)	30 (23.8)	
Stage (%)			0.422
Stage I	6 (60.0)	72 (59.5)	
Stage II	4 (40.0)	28 (23.1)	
Stage III	0 (0.0)	20 (16.5)	
Stage IV	0 (0.0)	1 (0.8)	

*BMI* body mass index

### Differential analysis of genes

Firstly, the difficultly detected mRNAs with read count value = 0 in more than 20% samples were filtered and deleted. Based on the read count of each sample, the DEGs in NAFLD compared to non-NAFLD were determined by DESeq2 with *p*-value <0.05 and |log_2_ fold change (FC)| > 1. With R package “pheatmap”, hierarchical clustering analysis of top 100 DEGs was conducted. ClusterProfiler (version 3.10.1) was applied to perform GO and KEGG pathway enrichment analysis of DEGs. The threshold was set at *p*-value <0.05.

### Differential analysis of DNA methylation

COHCAP package in R was used to analyze differentially methylated sites between NAFLD and non-NAFLD. Methylated sites with a *β* value = N/A in more than 20% samples were filtered and deleted. The threshold of differentially methylated sites was set as |Δ*β*| > 0.1 and *p*-value <0.05. Then, DMGs and MDEGs were obtained as well. Overlapping down-regulated and hypermethylation genes were identified as hypermethylated-lowly expressed genes (Hyper-LGs). Similarly, overlapping up-regulated and hypomethylation genes were considered hypomethylated-highly expressed genes (Hypo­HGs).

### Protein­protein interaction (PPI) network and survival analysis

A PPI network of Hyper-LGs and Hyper-LGs was built using the STRING database. Consequently, the PPI network was visualized by Cytoscape. In addition, to further investigate the prognostic value of MDEGs, the clinical data of samples were downloaded from TCGA to obtain survival information. Survival and SurvMiner packages were used for survival analysis after the integration of survival information and expression matrix.

### Expression validation of Hyper-LGs and Hypo­HGs by RT-PCR

To validate the expression of Hyper-LGs and Hypo­HGs, in vitro RT-PCR was performed. A total of 7 NAFLD of HCC patients with cirrhosis and 6 non-NAFLD of HCC patients with cirrhosis were enrolled in this study. The blood samples from the above individuals were collected for RT-PCR. GAPDH and ACTB were used as internal reference. The study was approved by the ethics committee of the Second People’s Hospital of Liaocheng (2022-44). In addition, all individuals provided the informed consent of the patients and their families.

## Results

### Identification of DEGs

**Fig. 1:**
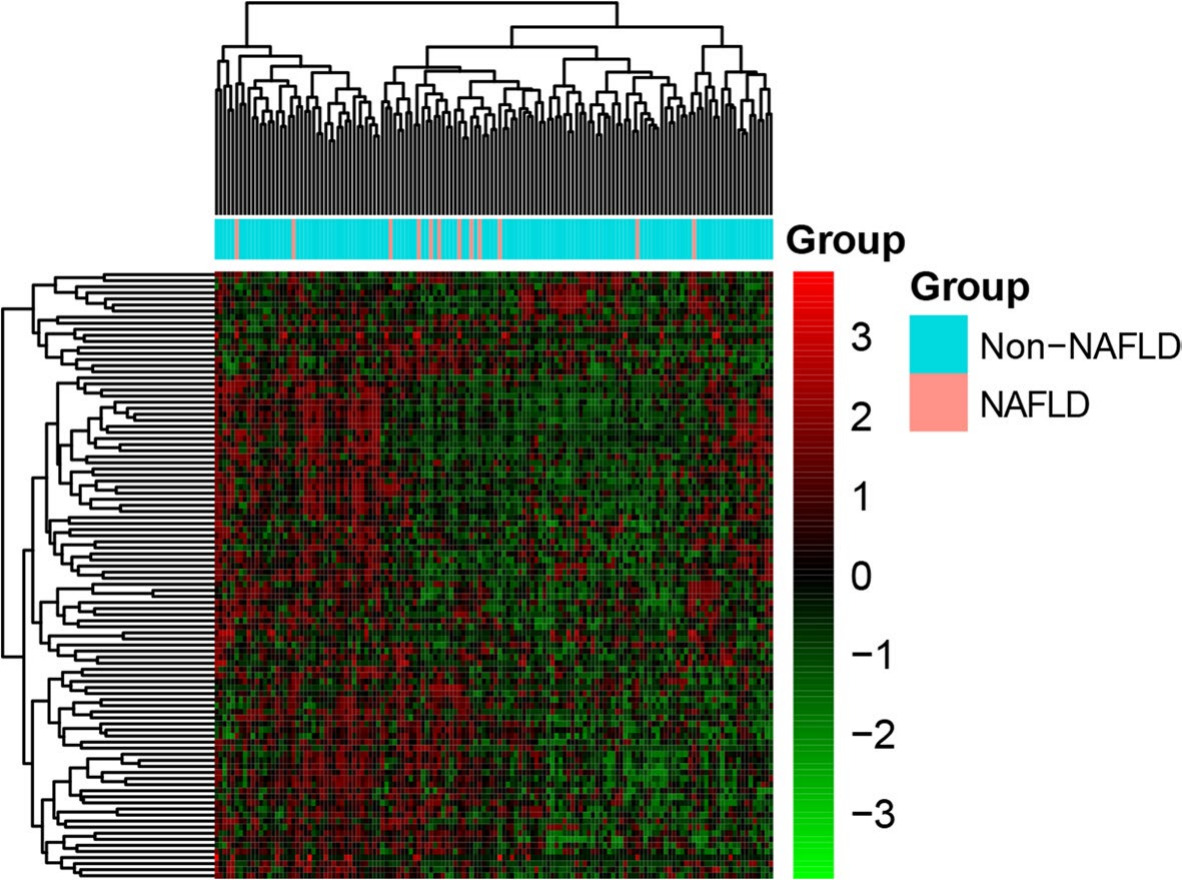
Hierarchical clustering analysis of top 100 DEGs. Row and column represented DEGs and samples, respectively. The color scale represented the expression levels

**Fig. 2:**
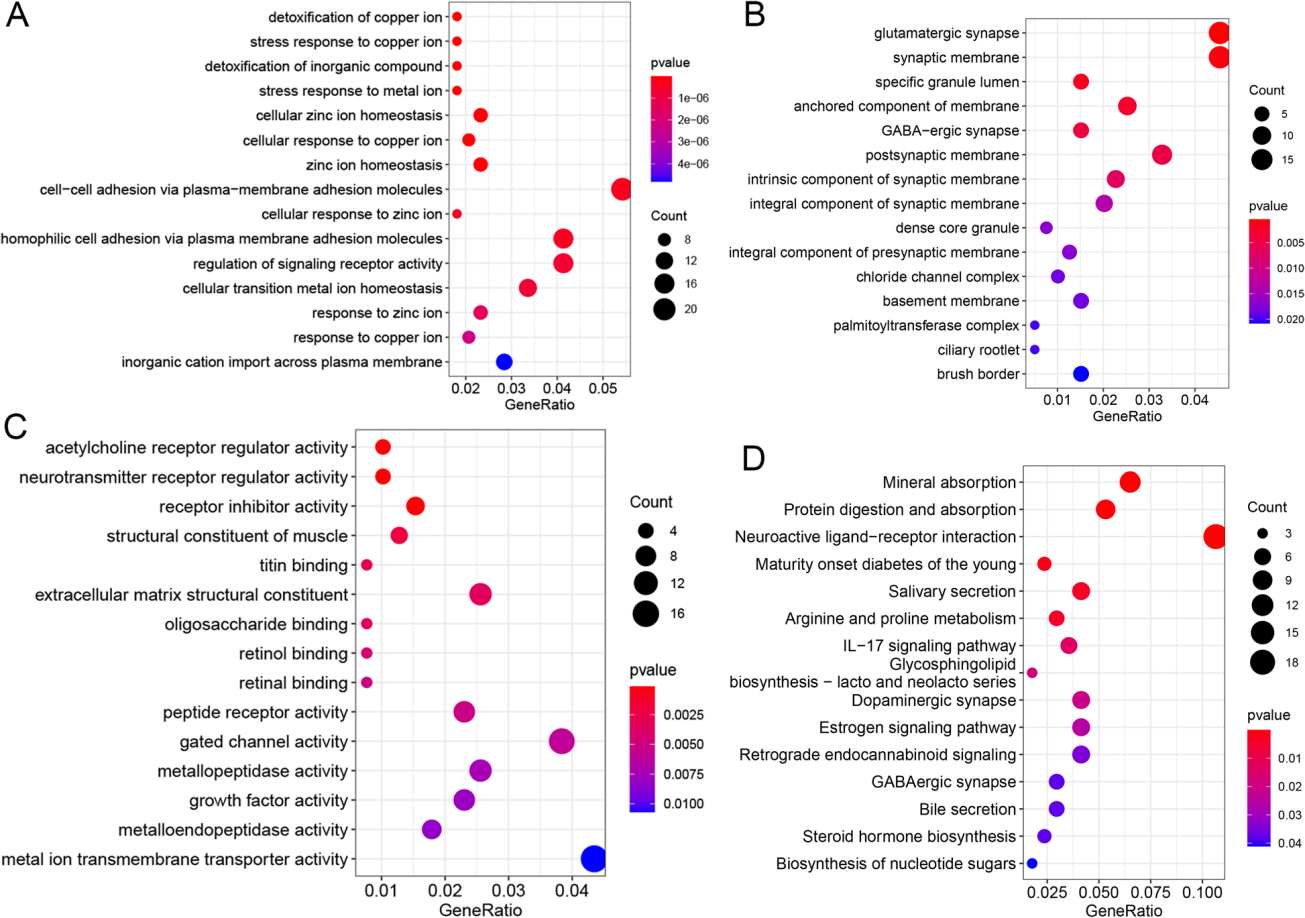
Significantly enriched GO terms and KEGG pathways of DEGs. **A** BP, biological process. **B** CC, cellular component. **C** MF, molecular function. **D** KEGG pathways

With *p*-value <0.05 and |log_2_FC| > 1, a total of 444 DEGs were identified in NAFLD, of which 79 genes (17.8%) were up-regulated while others were down-regulated. The heatmap of top 100 DEGs is shown in Fig. 1. Among them, PTCRA and MUC5B were the most up-regulated and down-regulated DEGs (Table 2). GO analysis indicated that DEGs were significantly enriched in detoxification of copper ion (*p*-value = 8.09E−09), glutamatergic synapse (*p*-value = 4.39E−04), acetylcholine receptor regulator activity (*p*-value = 1.25E−04), and neurotransmitter receptor regulator activity (*p*-value = 1.25E−04) (Fig. 2A-C). According to the KEGG pathway enrichment analysis, several pathways, including mineral absorption (*p*-value = 3.28E−08), protein digestion and absorption (*p*-value = 2.80E−04), and neuroactive ligand-receptor interaction (*p*-value = 3.82E−04) were significantly enriched (Fig. 2D).

**Table 2:** Top 10 up- and down-regulated DEGs

Symbol	log_**2**_FoldChange	***p***-value	Regulation
PTCRA	2.292772228	4.12E-06	up
SUCNR1	1.543556044	9.13E-06	up
HMP19	2.168629225	7.36E-05	up
HTR2A	1.88195227	8.77E-05	up
ADAM11	1.750207213	0.000184684	up
ERICH3	1.914312285	0.000273516	up
MSLN	1.653965567	0.000290518	up
ABCC11	1.320797175	0.000299271	up
KIT	1.241100576	0.000573308	up
PGR	1.853464361	0.001442046	up
MUC5B	-30	1.76E-135	down
TM4SF20	-30	6.05E-117	down
REG1B	-13.74638669	4.75E-14	down
MTRNR2L1	-5.976714996	1.85E-09	down
EPCAM	-5.012273559	4.65E-08	down
C6orf223	-4.948323683	7.42E-08	down
GNG4	-4.583539398	2.77E-07	down
ACTN2	-3.650444498	1.46E-06	down
NTS	-5.746044667	1.60E-06	down
REG1A	-5.811973564	1.81E-06	down

### Identification of DMGs

**Fig. 3:**
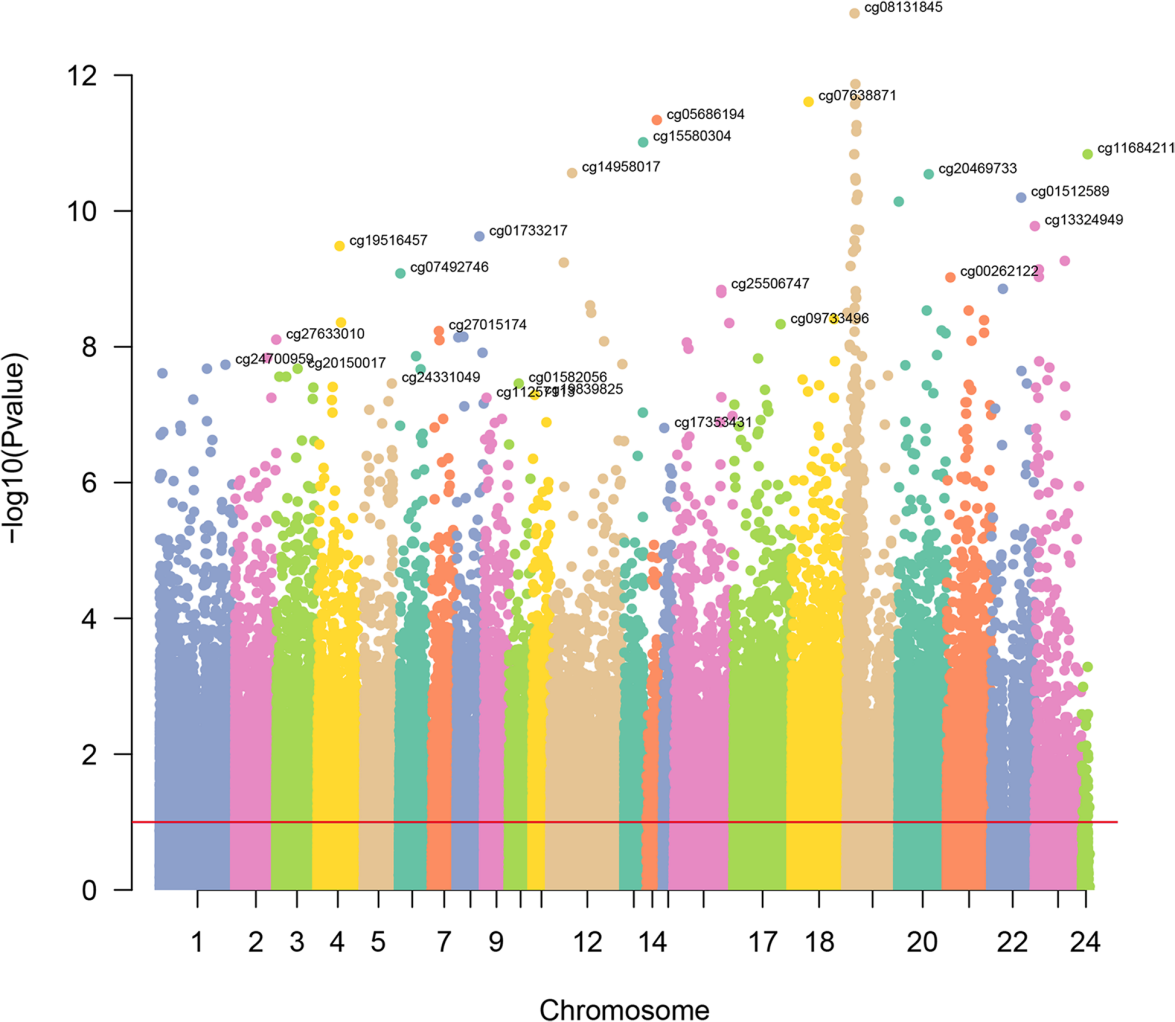
The Manhattan plot of differentially methylated sites in NAFLD. The *x*-axis represents the chromosome; the *y*-axis represents the −lg (*p*-value) of differentially methylated sites

Following the pre-treatment of methylation data without a *β* value, 395,042 sites were obtained. With the screening criteria of |Δ*β*| >0.1 and *p*-value <0.05, a total of 3082 differentially methylated sites and 1501 DMGs (481 hypermethylated genes and 1020 hypomethylated genes) were obtained. The Manhattan plot of these differentially methylated sites is presented in Fig. 3. By overlapping 79 up-regulated genes and 1020 hypomethylated genes, we obtained 5 Hypo­HGs (HOXD9, RAI2, ADPRHL1, C12orf42 and PCDHB16). By overlapping 365 down-regulated genes and 481 hypermethylated genes, we identified 13 Hyper-LGs (EPCAM, GNG4, SLFN13, USH1C, SPINT1, SLC39A4, LYZ, SPARCL1, DGKK, WNK2, DNAH9, STRA8 and ST8SIA3).

### PPI network and survival analysis

**Fig. 4:**
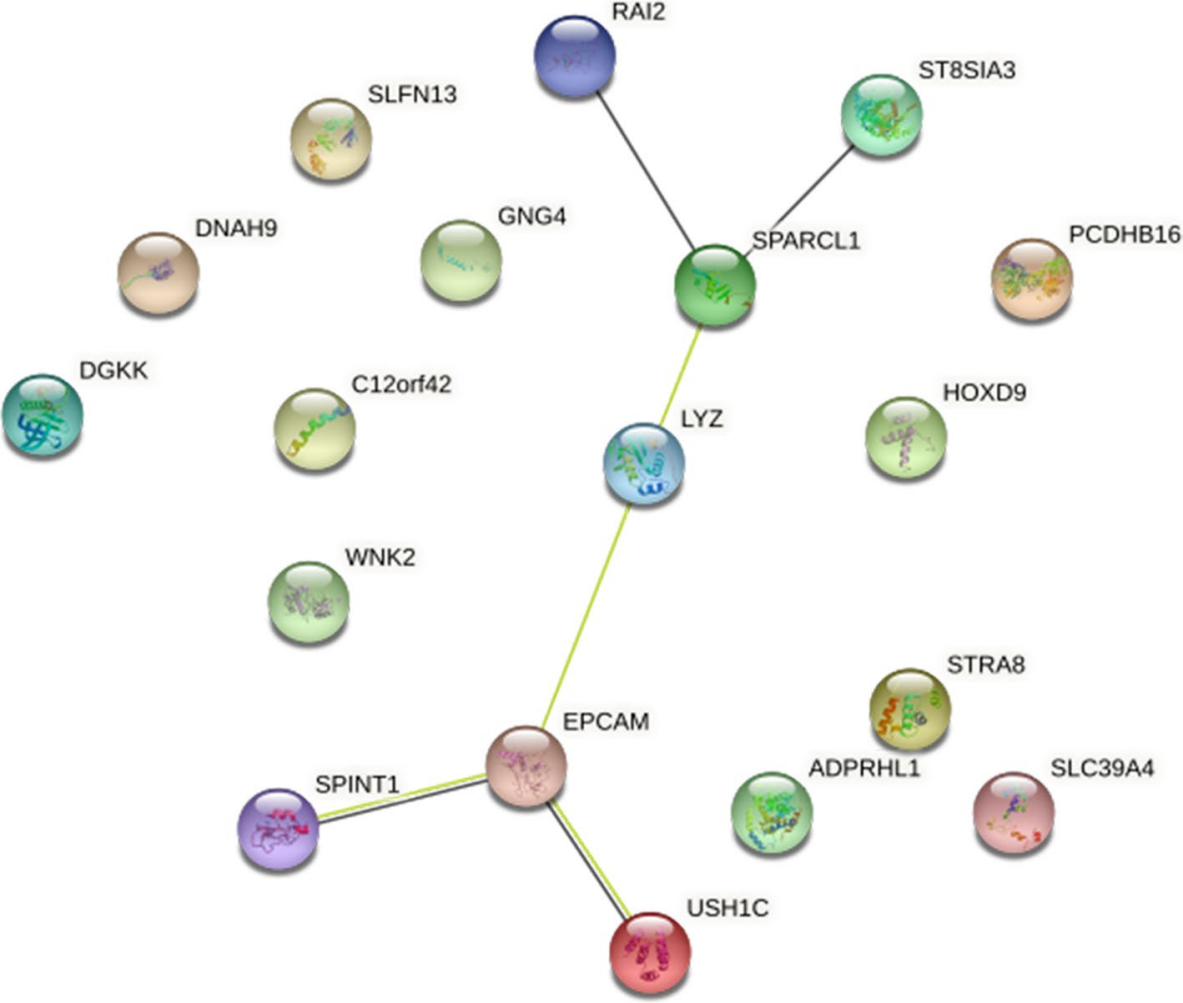
PPI network

**Fig. 5:**
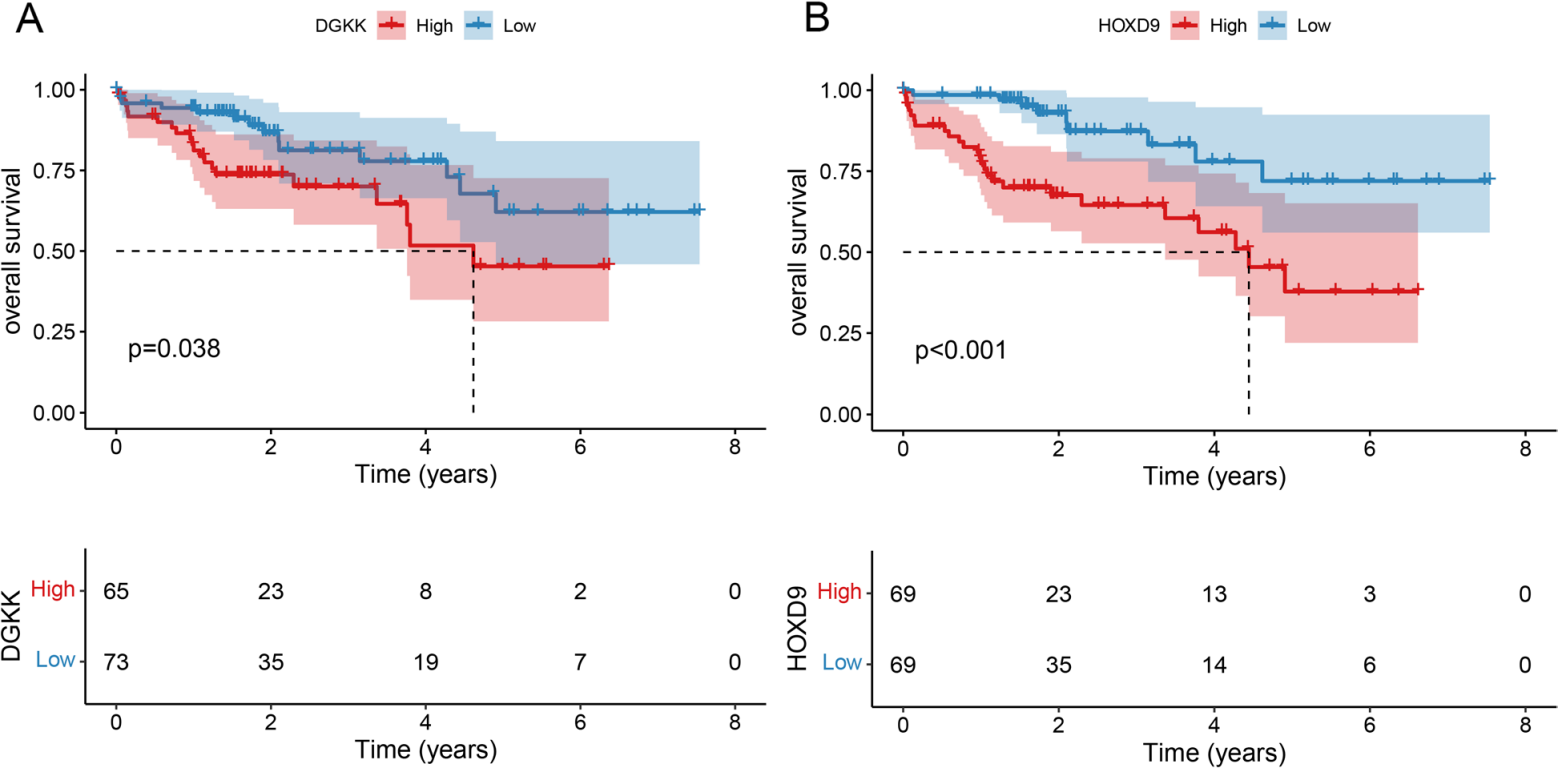
Survival analysis of MDEGs. **A** DGKK. **B** HOXD9. The *x*-axis indicated time (years) and *y*-axis indicated survival rate

With STRING online database and Cytoscape software, the PPI networks were constructed. PPI networks included 18 proteins, which consisted of two subgroups: 6 proteins with strong connections with others and 12 separated proteins (Fig. 4). EPCAM and SPARCL1 were two hub genes in PPI network. Survival analysis of these 18 MDEGs was performed, and the results indicated that the expression of DGKK (*p*-value = 0.038) and HOXD9 (*p*-value <0.001) was significantly correlated with the overall survival time of NAFLD patients (Fig. 5).

### Expression validation of Hyper-LGs and Hypo­HGs by RT-PCR

**Fig. 6:**
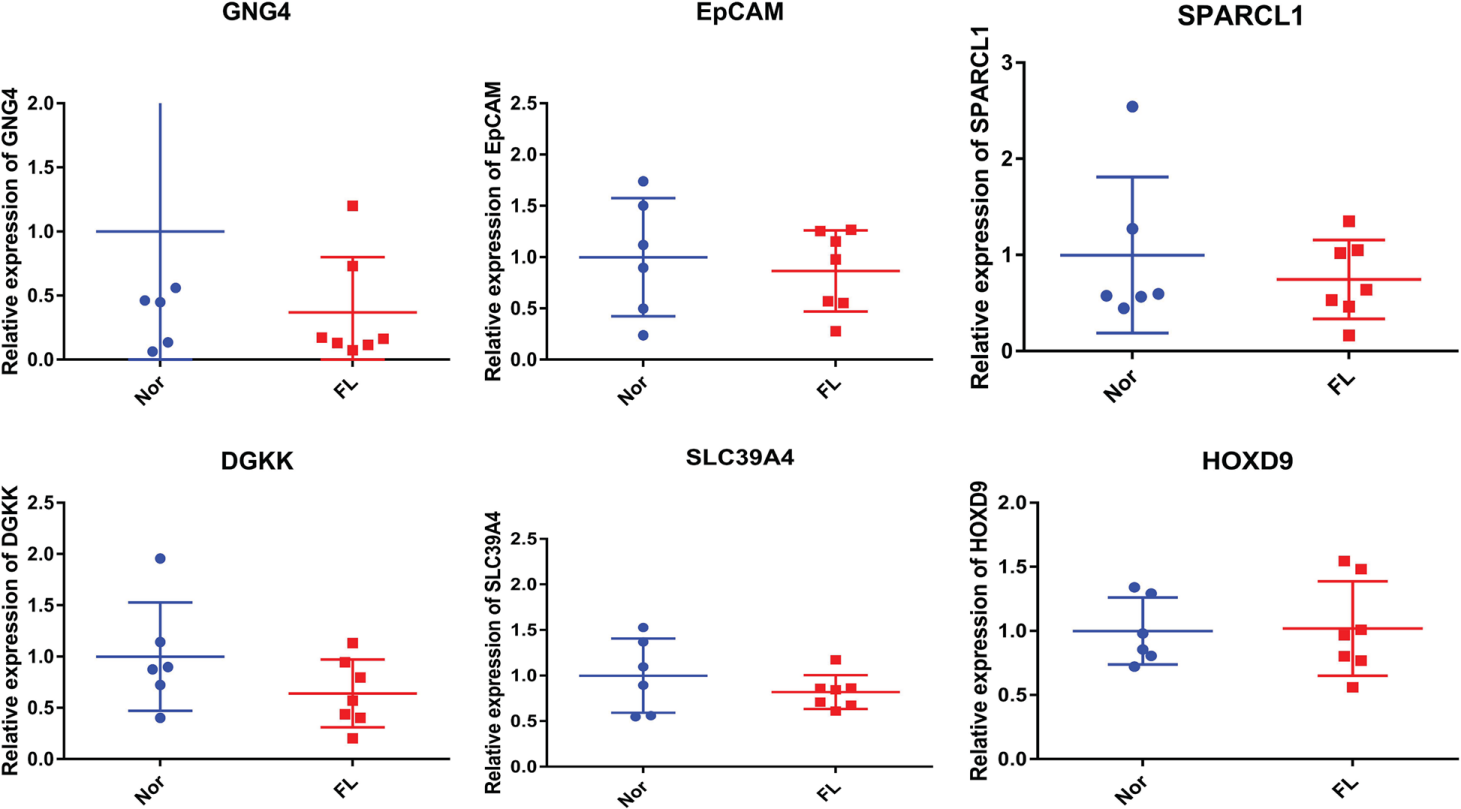
Expression validations of GNG4, EPCAM, SPARCL1, DGKK, SLC39A4, and HOXD9 by RT-PCR

To validate the expression of 5 Hyper-LGs (GNG4, EPCAM, SPARCL1, DGKK, and SLC39A4) and 1 Hypo­HGs (HOXD9), in vitro RT-PCR was performed in blood samples from 7 NAFLD of HCC patients with cirrhosis and 6 non-NAFLD of HCC patients with cirrhosis (Fig. 6). The clinical information of these individuals is listed in Table 3. Compared to non-NAFLD of HCC patients with cirrhosis, GNG4, EPCAM, SPARCL1, DGKK, and SLC39A4 were down-regulated and HOXD9 was up-regulated in NAFLD of HCC patients with cirrhosis. The expression trend of these genes was consistent with the bioinformatics analysis.

**Table 3:** The clinical information of NAFLD/non-NAFLD of HCC patients with cirrhosis

Number	Age	Gender	Drinking history	Smoking history	Family history	HBV infection	HCV infection	AFP (400μg/L)	Classification	Staging
NAFLD	50	Male	Yes	Yes	No	Positive	Negative	1600	2	IIA
NAFLD	60	Male	No	Yes	No	Negative	Negative	1700	2	IIB
NAFLD	59	Male	Yes	Yes	No	Negative	Negative	<400	3	III
NAFLD	54	Male	Yes	No	No	Negative	Negative	680	3	IV
NAFLD	57	Male	No	No	No	Negative	Negative	<400	4	IV
NAFLD	65	Female	No	No	No	Negative	Negative	<400	4	IIB
NAFLD	43	Female	No	No	No	Positive	Negative	635	1	III
Non-NAFLD	47	Male	Yes	Yes	No	Positive	Negative	720	4	IIA
Non-NAFLD	66	Male	No	No	No	Positive	Negative	<400	4	III
Non-NAFLD	60	Male	Yes	Yes	No	Positive	Negative	480	4	IIA
Non-NAFLD	43	Female	No	No	No	Positive	Negative	<400	3	IIB
Non-NAFLD	62	Female	No	No	No	Positive	Negative	1230	2	IV
Non-NAFLD	65	Female	Yes	No	No	Positive	Negative	630	1	IIB

*HBV* hepatitis B virus, *HCV* hepatitis C virus, *AFP* alpha fetal protein

## Discussion

It has been widely recognized that aberrant DNA methylation is significantly associated with HCC. In the present study, using the TCGA database, we searched for the candidates of cancer-related genes whose expressions are regulated by DNA methylation of NAFLD of HCC with cirrhosis.

High levels of GNG4 were reported in primary gastric cancer tissues as well as liver metastatic lesions, which were associated with short overall survival (OS) and the likelihood of liver recurrence [32]. It has been reported that GNG4 is up-regulated in colorectal carcinoma and liver metastases from colorectal carcinoma tissues, which is related to the OS and tumor-free survival of colorectal carcinoma patients [41]. Increased GNG4 expression is related to the poor prognosis and hypoxic microenvironment in lung adenocarcinoma [42]. Pal et al. reported that the promoter region of GNG4 was significantly hypermethylated and that its transcript level was significantly down-regulated in glioblastoma and renal cell carcinoma [23]. Mao et al. indicated that GNG4 was hypermethylated and its mRNA expression was significantly decreased in breast cancer [20]. In this study, GNG4 was significantly hypermethylated and one of the top 10 significantly down-regulated genes in NAFLD HCC patients with cirrhosis, suggesting that GNG4 may be a potential clinical therapeutic target for NAFLD HCC patients with cirrhosis by inhibiting tumor metastasis.

Epithelial cell adhesion molecule (EpCAM) is a type I transmembrane glycoprotein, acting as a Ca^2+^-independent homophilic cell adhesion molecule [13]. EpCAM has been reported to be involved in malignant proliferation, invasion, metastasis, and tumor recurrence [1]. Elevated EpCAM has been detected in various human tumors, including HCC [37]. The expression of EpCAM was significantly associated with inflammation in HBV infection, which serves as an early biomarker for HCC [4]. EpCAM has been demonstrated to be one of the targets of chemoresistance in human hepatocellular carcinoma cell lines [12]. At present, EPCAM was a hub gene in PPI network and one of the top 10 DEGs, as well as Hyper-LG, in NAFLD HCC patients with cirrhosis, which indicated that EPCAM may exert a momentous role in inflammatory response and tumorigenesis in NAFLD of HCC with cirrhosis. Maybe, EPCAM can be considered as a target of drug in the treatment of NAFLD of HCC with cirrhosis.

SPARCL1 has been reported to be expressed in confluent endothelial cells and is one of the signature genes for tumor angiogenesis [34]. Zhang et al. indicated that SPARCL1 was a prognostic biomarker in colorectal cancer and likely played a more significant role in the metastasis of primary colorectal cancer cells to normal liver tissues [39]. Liu et al. demonstrated that SPARCL1 was highly up-regulated in adipose tissue and played a role in exacerbating NASH progression in a mouse model of NASH [14]. Gao et al. suggested that SPARCL1 with AUC greater than 90% could be used as a diagnostic biomarker for liver cancer [6]. In this analysis, SPARCL1 was a hub gene in PPI network and Hyper-LG in NAFLD HCC patients with cirrhosis, which reminds us to focus on the role of tumor angiogenesis and metastasis of SPARCL1 in NAFLD of HCC with cirrhosis.

The DGKK gene (OMIM *300837), located on chromosome Xp11.22, encodes the diacylglycerol kinase kappa [8]. This enzyme is involved in the down-regulation of diacylglycerol signaling since it phosphorylates diacylglycerol, converting it to phosphatidic acid [27]. Genetic variants in DGKK have been strongly associated with risk for hypospadias [26]. In addition, up-regulated DGKK proteins are detected in HCC tumor tissue samples from mice treated with high-dose ascorbate [40]. Apart from this study, DGKK has rarely been reported in HCC. It is noted that DGKK was significantly correlated with overall survival time of NAFLD patients, which indicated that DGKK could be regarded as a potential prognostic marker molecule for NAFLD of HCC with cirrhosis patients.

Numerous studies have reported the highly expressed HOXD9 in HCC. It has been demonstrated that HOXD9 was strongly expressed and functioned as an oncogene to promote epithelial-mesenchymal transition and cancer metastasis in HCC [19]. Long et al. indicated that high expression levels of HOXD9 were relevant to a poor prognosis in HCC patients [17]. Over expressed HOXD9 was detected in HCC patients with microvascular invasion compared to patients without microvascular invasion and associated with poorer prognosis [35]. In addition, high level of HOXD9 has been closely linked to metastasis rate and poor prognosis in cervical cancer patients [36]. Previous studies have revealed that HOXD9 promoter methylation is higher in tumors than in healthy tissue and that DNA methylation levels correlate with the expression of HOXD9 mRNA and protein in malignant melanoma and glioma [21, 31]. Consistent with previous studies, HOXD9 was significantly associated with prognosis of NAFLD patients in this analysis, indicating that HOXD9 may be an effective clinical therapeutic target for NAFLD of HCC with cirrhosis.

## Conclusions

In conclusion, 5 Hypo­HGs (HOXD9, RAI2, ADPRHL1, C12orf42, and PCDHB16) and 13 Hyper-LGs (EPCAM, GNG4, SLFN13, USH1C, SPINT1, SLC39A4, LYZ, SPARCL1, DGKK, WNK2, DNAH9, STRA8, and ST8SIA3) were identified in NAFLD patients HCC with cirrhosis. Among them, EPCAM and SPARCL1 were identified as two hub genes and DGKK and HOXD9 were significantly correlated with prognosis. These genes may be involved in the development of NAFLD-related HCC with cirrhosis, which may be used in the clinical therapeutic targets. However, there are limitations to our study. Firstly, the sample size of the RT-PCR is small. Larger numbers of tissue and blood samples are further needed. Secondly, the potential molecular mechanism of identified genes is needed to explore in the animal models or cell experiment.

## Data Availability

The data supporting the conclusions of this article is included within the article and is available from the corresponding author on reasonable request.

## Abbreviations

NAFLD: Nonalcoholic fatty liver disease
NASH: Nonalcoholic steatohepatitis
HCC: Hepatocellular carcinoma
BCLB: Bcl 2 like protein 10
DEG: Differentially expressed gene
DMG: Differentially methylated gene
FC: Fold change
MDEG: Methylated differentially expressed gene
Hyper-LG: Hypermethylated, lowly expressed gene
Hypo­HG: Hypomethylated, highly expressed gene
PPI: Protein­protein interaction
OS: Overall survival
EpCAM: Epithelial cell adhesion molecule
